# Some new results on convex sequences

**DOI:** 10.1186/s13660-017-1438-4

**Published:** 2017-07-14

**Authors:** Hüseyin Bor

**Affiliations:** Independent Consultant, P.O. Box 121, TR-06502 Bahçelievler, Ankara, Turkey

**Keywords:** 26D15, 40D15, 40F05, absolute summability, convex sequence, Minkowsky inequality, Hölder inequality

## Abstract

In the present paper, we obtained a main theorem related to factored infinite series. Some new results are also deduced.

## Introduction

Let $\sum a_{n}$ be a given infinite series with $(s_{n})$ as the sequence of partial sums. In [1], Borwein introduced the $(C,\alpha ,\beta)$ methods in the following form: Let $\alpha+\beta\ne -1,-2,\ldots$ . Then the $(C,\alpha,\beta)$ mean is defined by

1 $$ {u_{n}^{\alpha,\beta}}=\frac{1}{A_{n}^{\alpha+\beta}} \sum _{v=1}^{n}{A_{n-v}^{\alpha-1}} {A_{v}^{\beta}}s_{v}, $$

where

2 $$ {A_{n}^{\alpha+\beta}}= O \bigl(n^{\alpha+\beta} \bigr),\qquad {A_{0}^{\alpha+\beta}=1}\quad \mbox{and}\quad {A_{-n}^{\alpha+\beta}=0} \quad \mbox{for } n>0. $$

The series $\sum{a_{n}}$ is said to be summable ${ \vert {C},\alpha,\beta ,\sigma;\delta \vert }_{k}$, $k\geq1$, $\delta\geq0$, $\alpha+\beta>-1$, and $\sigma\in {R}$, if (see [2])

3 $$ \sum_{n=1}^{\infty}n^{\sigma(\delta k+k-1)} \frac{ \vert t_{n}^{\alpha,\beta} \vert ^{k}}{{n}^{k}}< \infty, $$

where ${t_{n}^{\alpha,\beta}}$ is the $(C,\alpha,\beta)$ transform of the sequence $(na_{n})$. It should be noted that, for ${\beta=0}$, the ${ \vert {C},\alpha,\beta ,\sigma;\delta \vert }_{k}$ summability method reduces to the ${ \vert {C},\alpha,\sigma;\delta \vert }_{k}$ summability method (see [3]). Let us consider the sequence $(\theta_{n}^{\alpha,\beta})$ which is defined by (see [4])

4 $$\begin{aligned} \theta_{n}^{\alpha,\beta}= \textstyle\begin{cases} \vert t_{n}^{\alpha,\beta} \vert , & \alpha=1,\beta>-1, \\ \max_{1\leq v\leq n} \vert t_{v}^{\alpha,\beta} \vert , & 0< \alpha< 1, \beta>-1. \end{cases}\displaystyle \end{aligned}$$

## The main result

Here, we shall prove the following theorem.

### Theorem

*If* $(\lambda_{n})$ *is a convex sequence* (*see* [5]) *such that the series* $\sum\frac{\lambda_{n}}{n}$ *is convergent and let* $(\theta _{n}^{\alpha,\beta})$ *be a sequence defined as in* (4). *If the condition*

5 $$\begin{aligned} \sum_{n=1}^{m}n^{\sigma(\delta k+k-1)} \frac{(\theta_{n}^{\alpha,\beta })^{k}}{n^{k-1}}=O(m) \quad\textit{as } {m\rightarrow\infty} \end{aligned}$$

*holds*, *then the series* $\sum a_{n} \lambda_{n} $ *is summable* ${ \vert {C},\alpha,\beta,\sigma;\delta \vert }_{k}$, $k\geq1$, $0\leq \delta<\alpha\leq1$, $\sigma\in{R}$, *and* $({\alpha+\beta +1})k-{\sigma(\delta k+k-1)}>1 $.

*One should note that*, *if we set* $\sigma=1$, *then we obtain a well*-*known result of Bor* (*see* [6]).

We will use the following lemmas for the proof of the theorem given above.

### Lemma 1

[4]

*If* $0<\alpha\leq1$, $\beta>-1$, *and* $1 \leq v \leq n$, *then*

6 $$\begin{aligned} \Biggl\vert {\sum_{p=0}^{v}A_{n-p}^{\alpha-1}A_{p}^{\beta}a_{p}} \Biggr\vert \leq \max_{1 \leq m \leq v} \Biggl\vert {\sum _{p=0}^{m} A_{m-p}^{\alpha-1}A_{p}^{\beta}a_{p}} \Biggr\vert . \end{aligned}$$

### Lemma 2

[7]

*If* $(\lambda_{n})$ *is a convex sequence such that the series* $\sum\frac{\lambda_{n}}{n}$ *is convergent*, *then* $n{\Delta\lambda_{n}}\rightarrow0\textit{ as }n\rightarrow\infty$ *and* $\sum_{n=1}^{\infty} (n+1)\Delta^{2} {\lambda_{n}}$ *is convergent*.

## Proof of the theorem

Let $(T_{n}^{\alpha,\beta})$ be the *n*th $(C,\alpha,\beta)$ mean of the sequence $(n{a_{n}}{\lambda_{n}})$. Then, by (1), we have

$$\begin{aligned} T_{n}^{\alpha,\beta} = & \frac{1}{A_{n}^{\alpha+\beta}} \sum _{v=1}^{n}A_{n-v}^{\alpha-1}A_{v}^{\beta} va_{v}\lambda_{v} . \end{aligned}$$

First applying Abel’s transformation and then using Lemma 1, we have

$$\begin{aligned} &T_{n}^{\alpha,\beta} = \frac{1}{A_{n}^{\alpha+\beta}} \sum _{v=1}^{n-1}\Delta\lambda_{v} \sum _{p=1}^{v}A_{n-p}^{\alpha-1}A_{p}^{\beta}p a_{p}+ \frac{\lambda_{n}}{A_{n}^{\alpha+\beta}} \sum_{v=1}^{n} {A_{n-v}^{\alpha-1}}A_{v}^{\beta}va_{v}, \\ &\bigl\vert T_{n}^{\alpha,\beta} \bigr\vert \leq \frac{1}{A_{n}^{\alpha+\beta}} \sum_{v=1}^{n-1} \vert { \Delta\lambda_{v}} \vert \Biggl\vert {\sum _{p=1}^{v}A_{n-p}^{\alpha-1}A_{p}^{\beta}p a_{p}} \Biggr\vert + \frac{ \vert \lambda_{n} \vert }{A_{n}^{\alpha+\beta}} \Biggl\vert \sum _{v=1}^{n} {A_{n-v}^{\alpha-1}} {A_{v}^{\beta}}v{a_{v}} \Biggr\vert \\ &\phantom{\bigl\vert T_{n}^{\alpha,\beta} \bigr\vert }\leq \frac{1}{A_{n}^{\alpha+\beta}} \sum_{v=1}^{n-1} A_{v}^{\alpha}A_{v}^{\beta} \theta_{v}^{\alpha,\beta} \vert {\Delta \lambda_{v}} \vert + \vert {\lambda_{n}} \vert \theta_{n}^{\alpha,\beta} \\ &\phantom{\bigl\vert T_{n}^{\alpha,\beta} \bigr\vert }= T_{n,1}^{\alpha,\beta} + T_{n,2}^{\alpha,\beta}. \end{aligned}$$

In order to complete the proof of the theorem by using Minkowski’s inequality, it is sufficient to show that

$$\sum_{n=1}^{\infty}n^{\sigma(\delta k+k-1)} \frac{ \vert T_{n,r}^{\alpha,\beta} \vert ^{k}}{n^{k}} < \infty,\quad \mbox{for } r=1,2. $$

For $k>1$, we can apply Hölder’s inequality with indices *k* and ${k'}$, where $\frac{1}{k}+\frac{1}{k'}=1$, and we obtain

$$\begin{aligned} \sum_{n=2}^{m+1}n^{\sigma(\delta k+k-1)} \frac{ \vert {T_{n,1}^{\alpha,\beta}} \vert ^{k}}{n^{k}} \leq{}&\sum_{n=2}^{m+1} n^{\sigma(\delta k+k-1)-k} \Biggl\vert { \frac{1}{A_{n}^{\alpha+\beta}} \sum _{v=1}^{n-1}A_{v}^{\alpha}A_{v}^{\beta} \theta_{v}^{\alpha,\beta}\Delta \lambda_{v}} \Biggr\vert ^{k} \\ = {}& O(1) \sum_{n=2}^{m+1} \frac{1}{n^{(\alpha+ \beta +1)k-\sigma(\delta k+k-1)}} \Biggl\{ \sum_{v=1}^{n-1} v^{\alpha k}v^{\beta k} \Delta\lambda_{v} \bigl( \theta_{v}^{\alpha,\beta} \bigr)^{k} \Biggr\} \\ &{}\times \Biggl\{ \sum_{v=1}^{n-1}\Delta \lambda_{v} \Biggr\} ^{k-1} \\ = {}& O(1)\sum_{v=1}^{m} v^{(\alpha+\beta) k} \Delta\lambda_{v} \bigl(\theta_{v}^{\alpha,\beta} \bigr)^{k} \sum_{n=v+1}^{m+1} \frac{1}{n^{(\alpha+ \beta+1)k-\sigma(\delta k+k-1)}} \\ = {}& O(1)\sum_{v=1}^{m}v^{(\alpha+\beta) k} \Delta\lambda_{v} \bigl(\theta_{v}^{\alpha,\beta} \bigr)^{k} \int_{v}^{\infty} \frac{dx}{ x^{(\alpha+ \beta+1)k-\sigma(\delta k+k-1)}} \\ = {}& O(1)\sum_{v=1}^{m}\Delta \lambda_{v}v^{\sigma(\delta k+k-1)} \frac{(\theta_{v}^{\alpha,\beta})^{k}}{v^{k-1}} \\ = {}& O(1)\sum_{v=1}^{m-1}\Delta(\Delta \lambda_{v})\sum_{p=1}^{v} p^{\sigma(\delta k+k-1)}\frac{(\theta_{p}^{\alpha,\beta})^{k}}{p^{k-1}} \\ &{}+ O(1)\Delta\lambda_{m}\sum_{v=1}^{m} v^{\sigma(\delta k+k-1)}\frac {(\theta_{v}^{\alpha,\beta})^{k}}{v^{k-1}} \\ ={} & O(1)\sum_{v=1}^{m} v \Delta^{2}\lambda_{v} + O(1)m {\Delta\lambda_{m}} = O(1) \quad\mbox{as } {m\rightarrow\infty}, \end{aligned}$$

by virtue of hypotheses of the theorem and Lemma 2. Similarly, we have

$$\begin{aligned} \sum_{n=1}^{m}n^{\sigma(\delta k+k-1)} \frac{ \vert {T_{n,2}^{\alpha,\beta}} \vert ^{k}}{n^{k}} = {}& O(1)\sum_{n=1}^{m} \frac {\lambda_{n}}{n}n^{\sigma(\delta k+k-1)}\frac{(\theta_{n}^{\alpha,\beta })^{k}}{n^{k-1}} \\ = {}& O(1)\sum_{n=1}^{m-1} \Delta \biggl( \frac{\lambda_{n}}{n} \biggr)\sum_{v=1}^{n}v^{\sigma(\delta k+k-1)} \frac{({\theta_{v}^{\alpha,\beta}})^{k}}{v^{k-1}} \\ &{} + O(1)\frac{\lambda_{m}}{m}\sum_{n=1}^{m}n^{\sigma(\delta k+k-1)} \frac{({\theta_{n}^{\alpha,\beta}})^{k}}{n^{k-1}} \\ = {}& O(1)\sum_{n=1}^{m-1}\Delta \lambda_{n} + O(1)\sum_{n=1}^{m-1} \frac{\lambda_{n+1}}{n+1} + O(1)\lambda_{m} \\ ={} & O(1)\sum_{n=1}^{m-1}\Delta \lambda_{n} + O(1)\sum_{n=2}^{m-1} \frac{\lambda_{n}}{n} + O(1)\lambda_{m} \\ = {}& O(1) (\lambda_{1}-\lambda_{m})+ O(1)\sum _{n=1}^{m-1}\frac{\lambda_{n}}{n} + O(1) \lambda_{m} \\ = {}&O(1) \quad\mbox{as } {m\rightarrow\infty} \end{aligned}$$

in view of hypotheses of the theorem and Lemma 2. This completes the proof of the theorem.

## Conclusions

By selecting proper values for *α*, *β*, *δ*, and *σ*, we have some new results concerning the ${ \vert {C,1} \vert }_{k}$, ${ \vert {C},\alpha \vert }_{k}$, and ${ \vert {C},\alpha ;\delta \vert }_{k}$ summability methods.
